# Janus Hydrogel Microparticles with Tea Polyphenol/Melanin Nanoparticles Integration from Microfluidics for Wound Healing

**DOI:** 10.1002/smsc.202500322

**Published:** 2025-08-27

**Authors:** Zhiqiang Luo, Danqing Huang, Yuanjin Zhao

**Affiliations:** ^1^ Department of Rheumatology and Immunology Nanjing Drum Tower Hospital School of Biological Science and Medical Engineering Southeast University Nanjing 210096 P. R. China; ^2^ Wenzhou Institute University of Chinese Academy of Sciences Wenzhou Zhejiang 325001 P. R. China

**Keywords:** Janus, melanin, microfluidics, particles, tea polyphenol, wounds

## Abstract

Massive bioactives show outstanding performance in promoting wound healing, while their combined application remains challenging in avoiding mutual interference. Herein, Janus hydrogel microparticles co‐loaded with tea polyphenol‐magnesium and melanin nanoparticles for synergistically antioxidant and antibacterial wound treatment are proposed. The tea polyphenol‐magnesium nanoparticles are prepared by the self‐assembly of tea polyphenol molecule and magnesium ion, while melanin nanoparticles are harvested from black hair. These two kinds of nanoparticles are, respectively, embedded into the hemisphere of Janus microparticles by microfluidics technology. The in vitro tests reveal that the resultant Janus microparticles have high biocompatibility and show enhanced antioxidant performance and synergistical antibacterial activity. Additionally, when the Janus microparticles are applied to the treatment of diabetic wounds, they promote the wound healing by scavenging reactive oxygen species, killing bacteria, reducing inflammation, decreasing matrix metalloproteinase‐9 secretion, and enhancing tissue remolding. Therefore, it is believed that the Janus microparticles with melanin and tea polyphenol‐Mg nanoparticles are highly promising candidate for clinical wound healing.

## Graphical abstract

Tea polyphenol‐magnesium nanoparticles are prepared by the self‐assembly of tea polyphenol and magnesium ion. Melanin nanoparticles are harvested from black hair. Janus hydrogel microparticles co‐loaded with tea polyphenol‐magnesium and melanin are fabricated for synergistically antioxidant and antibacterial. They promote the diabetic wound healing through antioxidant, antibacterial, and decreasing matrix metalloproteinase‐9 secretion.

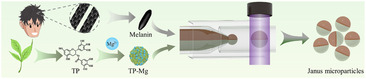

## 1 Introduction

Wound management is a worldwide healthcare issue.^[^ ^1^, ^2^, ^3^, ^4^ ^]^ Generally, the wound healing process is associated with inflammation, physiological signal response, tissue remodeling, etc.^[^ ^5^, ^6^, ^7^ ^]^ This comprehensive process is typically delayed, particularly in patients with conditions such as diabetes, due to their weak immune‐protective ability against threats.^[^ ^8^, ^9^, ^10^, ^11^, ^12^ ^]^ Among them, endogenous factors primarily pose threats through excessive reactive oxygen species (ROS), abnormal extracellular matrix (ECM) deposition, and disordered physiological signal responses; while exogenous factors mainly refer to bacterial infection.^[^ ^13^, ^14^, ^15^, ^16^ ^]^ To combat threat factors and promote wound healing process, various bioactive substances and therapeutic strategies have been proposed, such as melanin for clearing ROS, tea polyphenol (TP) for destroying biofilm, photothermal nanomaterials for killing bacteria. and so on.^[^ ^17^, ^18^, ^19^, ^20^, ^21^, ^22^ ^]^ Although these treatments are effective in their own way, in wounds with complex pathological factors, the combination of multiple therapies may achieve maximum curing effect. However, when multiple drugs are simply mixed together, the performance of their functions may be hampered by the occurred unwanted mutual reactions. Therefore, it is essential to develop a method to effectively integrate multiple therapeutics for complex wounds treatment.

**Figure 1:**
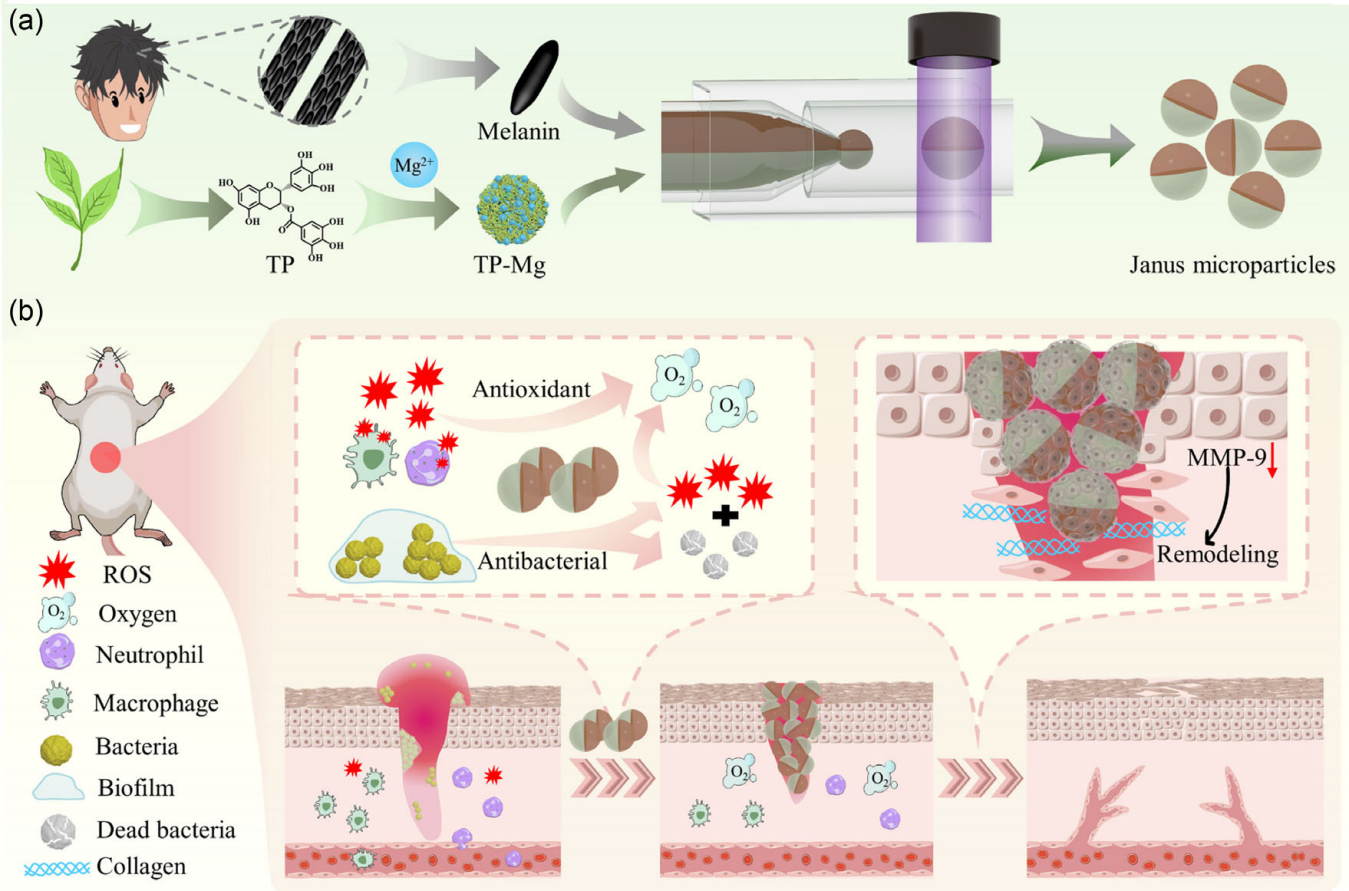
Schematic diagram of multiactives‐loaded Janus microparticles for infected wound healing. a) Melanin nanoparticles extracted from black hair and synthesized TP‐Mg nanoparticles for Janus hydrogel microparticle fabrication via microfluidic technology. b) The Janus microparticles promote infected wound healing through destroying biofilms, scavenging ROS, eliminating bacteria in the early healing stage, as well as enhancing regenerated tissues remodeling and improving collagen deposition at final stage.

In this article, we proposed an innovative Janus hydrogel microparticle with spatially separated multiactives via microfluidics for complex wound healing, as illustrated in **Figure** **1**. Hydrogel microparticles with designed architectures have shown great advantages in multidrug delivery, especially Janus structure.^[^ ^23^, ^24^, ^25^, ^26^, ^27^, ^28^, ^29^ ^]^ Janus microparticles refer to microspheres with comparted structures, where one hemisphere has different properties than the other.^[^ ^30^, ^31^ ^]^ By loading different actives in each hemisphere, Janus microparticle can realize multiple drug delivery with avoided unwanted reactions and increased drug stability. In contrast, microfluidic technology provides significant advantages in precise fluid administration and on‐demand components integration, showing potential in manufacturing Janus microparticles with tunable structure and tailored components.^[^ ^32^, ^33^, ^34^, ^35^ ^]^ Considering the synergetically antioxidant and antibacterial effect of melanin and TP, it is anticipated that microfluidic Janus microparticles with comparted loading of these two actives can achieve synergistic effect and reduce potential side impact during complex wound treatment.

Here, extracted melanin nanoparticles from black hair and synthesized tea polyphenol‐magnesium (TP‐Mg) nanoparticles were incorporated into Janus hydrogel microparticles by microfluidics for diabetic wound healing. These melanin nanoparticles presented near‐inferred (NIR)‐responsive heating performance and stable antioxidant effect in wide pH conditions. TP‐Mg nanoparticles that obtained by the reaction of TP and Mg in the alkaline environment can destroy biofilms to kill bacteria and also scavenge free radicals. After integration by microfluidics, the melanin and TP‐Mg co‐loaded Janus microparticles were endowed with synergetically antibacterial and antioxidant properties with ideal stability and biocompatibility. When applied to the treatment of bacteria‐infected diabetic wounds, the Janus microparticles can significantly clear ROS and eliminate bacteria in the early healing stage. Additionally, they can also enhance the remodeling of regenerated tissues by downregulating inflammatory response, reducing MMP‐9 secretion, improving collagen deposition, and enhancing revascularization. Therefore, we believed that our Janus microparticle with two nanoparticles integration is a prospective therapeutic strategy for complex wound treatment in clinic.

## 2 Results and Discussion

**Figure 2:**
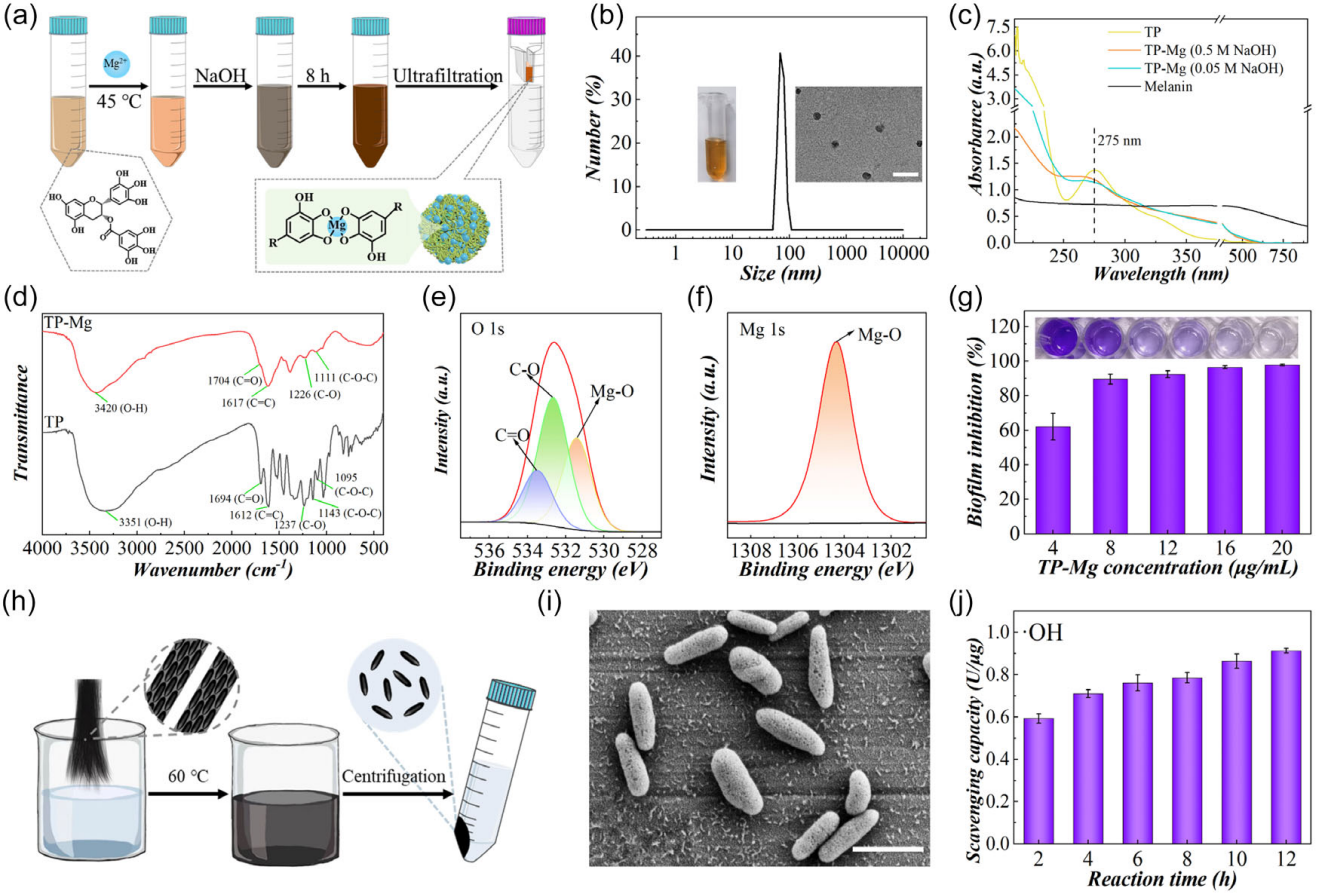
Fabrication and characterization of melanin and TP‐Mg nanoparticles. a) The schematic of TP‐Mg fabrication. b) The hydrodynamic size of TP‐Mg nanoparticles detected by DLS. The inserted images are photos of the final TP‐Mg nanoparticles suspension and their TEM image. c) The UV–vis spectrum of TP, TP‐Mg nanoparticles, and melanin nanoparticles. d) The FTIR spectrum of TP and TP‐Mg nanoparticles. e,f) The XPS spectrum of TP‐Mg nanoparticles. g) The inhibition capacity of TP‐Mg nanoparticles to biofilm formation. h) The schematic of melanin nanoparticles extraction. i) The SEM image of melanin nanoparticles. j) The ·OH scavenging capacity of melanin nanoparticles fabricated from different parameters. Scale bars are 200 nm in (b) and 1 μm in i). Sample size *n* = 3 for all experiments.

In a typical experiment, TP‐Mg nanoparticles were synthesized by a two‐step reaction with optimized parameters (**Figure** **2**a). TP is a mixture of organic polyhydroxyl compounds. Under the alkaline environment, the phenolic hydroxyl of TP can form complex with Mg^2+^, forming stable nanoparticles. The fabricated TP‐Mg nanoparticles were washed in ultrafiltration tube by centrifuging to purify the products. It is worth noting that the TP can also self‐assemble to form nanoparticles in the weak alkaline environment. Under these two actions, there may be not uniform nanoparticles in the reaction systems, influenced by both of the sodium hydroxide (NaOH) concentration and Mg^2+^ content. In all the tested parameters, the main nanoparticles products that could not be stably suspended were fabricated when the Mg^2+^ content reached 0.1 mmol and the concentration of added NaOH solution reached 5 M (Figure S1, Supporting Information). Considering the impediment of suspension instability to following microfluidics integration, their investigations were not further conducted. Uniformed and stable suspended nanoparticles can be fabricated at the parameter of 0.05 mmol Mg^2+^ and 0.5 M NaOH. Dynamic light scattering (DLS) result and image from transmission electron microscopy (TEM) showed that the fabricated TP‐Mg nanoparticles had good dispersion, at a size of near 70 nm (Figure 2b).

When the NaOH concentration was equal to or less than 0.05 M, the solution color changed gradually and there were two kinds of nanoparticles in the final products, at the hydrodynamic size of lower than 10 and near 100 nm, respectively (Figure S2a and c, Supporting Information). In contrast, the color of reacting solution was dramatically changed once the 0.5 M NaOH solution was dropped into the TP and Mg^2+^ solution (Figure S2b, Supporting Information). Meanwhile, with the decrease of Mg content, the change of nanoparticles size was little (Figure S2d, Supporting Information). Fourier transform infrared (FTIR) spectrum of the nanoparticles fabricated from the parameter of lower than 0.5 M NaOH presented the great similarity with TP, thus, the main products were referred to be the self‐assembled TP‐related nanoparticles mixture (Figure S3a, Supporting Information). In contrast, FTIR spectrum of the uniformed nanoparticles presents the great fluence of Mg to O‐related groups after forming complex, including C—O and C=O bond of phenolic hydroxyl group, ether group, and ester group (Figure 2d). After the formation of complex, the absorption peak of phenolic hydroxyl group was obviously changed as well, as revealed by ultraviolet (UV)‐via spectrum (Figure 2c). The data from X‐Ray photoelectron spectrometer (XPS) also supported the existence of Mg—O in the final products (Figure 2e, f and S3b, Supporting Information). All of these results demonstrated the successful fabrication of TP‐Mg nanoparticles.

The inhibition property to bacteria proliferation was selected as the main factor to optimize the most curative TP‐related nanoparticles. It is found that the TP‐Mg nanoparticles from 0.05 mmol Mg^2+^/0.5 M NaOH presented the better inhibition capacity (Figure S3c and e, Supporting Information). Meanwhile, their antioxidant property that was relying on phenolic hydroxyl group was also quantified. The results revealed that the uniform TP‐Mg nanoparticles were also imparted with higher OH scavenging activity than the other parameters (Figure S3d and f, Supporting Information). Based on those data, the TP‐Mg nanoparticles from the parameter of 0.05 mmol Mg^2+^ and 0.5 M NaOH were used for further test due to high inhibition of bacteria proliferation and antioxidant property. As the derivative of TP, TP‐Mg nanoparticles were supposed to present the biofilm destruction property of TP, which was subsequently proved. With the increase of TP‐Mg nanoparticles concentration, the formation of biofilm was significantly inhibited. The TP‐Mg nanoparticles concentration of 12 μg mL^−1^ can achieve the over 80% inhibition of biofilms formation (Figure 2g). For the mature biofilm, it could also be destroyed by TP‐Mg nanoparticles (Figure S3g, Supporting Information). These results indicated that TP‐Mg nanoparticles were imparted with the antioxidant by scavenging ·OH and antibacterial by destroying biofilm and inhibiting proliferation.

Melanin was harvested from black hair through reaction with NaOH. Black hair is mainly consisted of melanin and keratin. When keratin was dissolved by NaOH solution, melanin nanoparticles can be obtained (Figure 2h and S4a, Supporting Information). The obtained melanin nanoparticles showed rod‐like morphology with nanopores (Figure 2i). Our previous research has reported its free radical‐scavenging capacity with both superoxide dismutase‐like and catalase‐like nanoenzyme activity, which was selected as the main factor to optimize the parameters of fabrication procedure.^[^ ^17^ ^]^ With the prolong of reaction time, it is found that the ·OH scavenging capacity of obtained melanin particles was enhanced (Figure 2j). The concentration of NaOH also influenced the antioxidant property of obtained melanin nanoparticles but the change was less significant (Figure S4b, Supporting Information). In all the tested conditions, the morphology of the harvested nanoparticles kept the rod‐like shape (Figure S5a‐b, Supporting Information). The melanin nanoparticles from the parameter of 12 h reaction time and 1 M NaOH were chosen for following test due to high antioxidant and lower cost. The fabricated melanin nanoparticles showed the property of full‐wavelength light absorption (Figure 2c). Especially, it is found that they could absorb the NIR light to generate heat, arising the increase of local temperature. This NIR‐induced heating hurt has been reported to be an effective collaborative antibacterial method that can be combined with other strategies.^[^ ^36^ ^]^ The heating efficiency of melanin nanoparticles was depended on the concentration of melanin suspension and laser power of NIR light. Specifically, the temperature of melanin suspension increased with the increase of melanin concentration at the same NIR power and the increase of NIR power at the same melanin concentration (Figure S5c‐d, Supporting Information). These results demonstrated that melanin nanoparticles were endowed with the antioxidant of nanoenzyme activities and antibacterial by NIR‐induced heating hurt to bacteria.

pH value is varied in the different wounds and different healing periods, which is an important factor to influence the ROS‐scavenging efficiency of bioactives. Melanin nanoparticles act as nanoenzymes with both superoxide dismutase‐like and catalase‐like enzyme activity to eliminate free radical while TP‐Mg nanoparticles can scavenge free radical through their phenolic hydroxyl group. To further assess the comprehensive antioxidant capacity of fabricated two nanoparticles, 2‐Phenyl‐4,4,5,5‐tetramethylimidazoline‐3‐oxide‐1‐oxyl (PTIO) and Diphenylpicrylhydrazyl (DPPH) were used as free radical models of reactive oxide species (ROS) and reactive nitrogen species, respectively. DPPH was tested in alcohol environment while PTIO with higher chemical stability was tested in different pH solutions. The data indicated that the scavenging rates were rising with the increase of melanin nanoparticles and TP‐Mg nanoparticles (Figure S6a‐b, Supporting Information). The nanoparticles concentration of 0.4 mg mL^−1^ melanin suspension could scavenge over 80% free radical while 50 μg mL^−1^ TP‐Mg nanoparticles suspension could scavenge near 86% free radical. The trends of PTIO scavenging kept the same with the results of DPPH test at all the tested pH conditions, proving the stable antioxidant capacities of two kinds of nanoparticles (Figure S6c‐d, Supporting Information).

To administrate these nanoparticles, methylacrylic anhydride (MA)‐modified collagen (ColMA)‐based Janus hydrogel microparticles were developed to achieve better curative results. ColMA was synthesized by reaction between collagen and MA.^[^ ^37^ ^]^ After modification, the obtained ColMA retained the typical amide structure of collagen without obvious ester bond, indicating that the modification site is amino groups of collagen, as confirmed by FTIR spectrum (Figure S7a, Supporting Information). Under UV exposure, its sol solution can be polymerized into hydrogel (Figure S7b, Supporting Information). Taking ColMA as main matrix, Janus hydrogel microparticles were manufactured in a microfluidics chip constructed by θ tube and round capillary, which was coaxially arranged on glass slide (Figure S8a, Supporting Information). The ColMA solution dispersed with nanoparticles was used as the precursor of microfluidics chip. The two‐precursor fluid flowed along the channels of θ tube and contacted at the nozzle, forming laminar flow and continuing to flow. Under the shearing of outer fluid, microdroplets formed and prepolymerized under UV point light for easy collection (Figure S8b, Supporting Information). The size of manufactured droplets was uniform and could be adjusted by changing the flow rates of inner phase and outer phase (Figure S8c, Supporting Information). In specific, at the constant flow rate of inner phase, the size of microdroplets decreased with the increase of flow rate of outer phase. At the constant flow rate of outer phase, the diameter of microdroplets decreased with the decrease of flow rate of inner phase (Figure S8d, Supporting Information).

**Figure 3:**
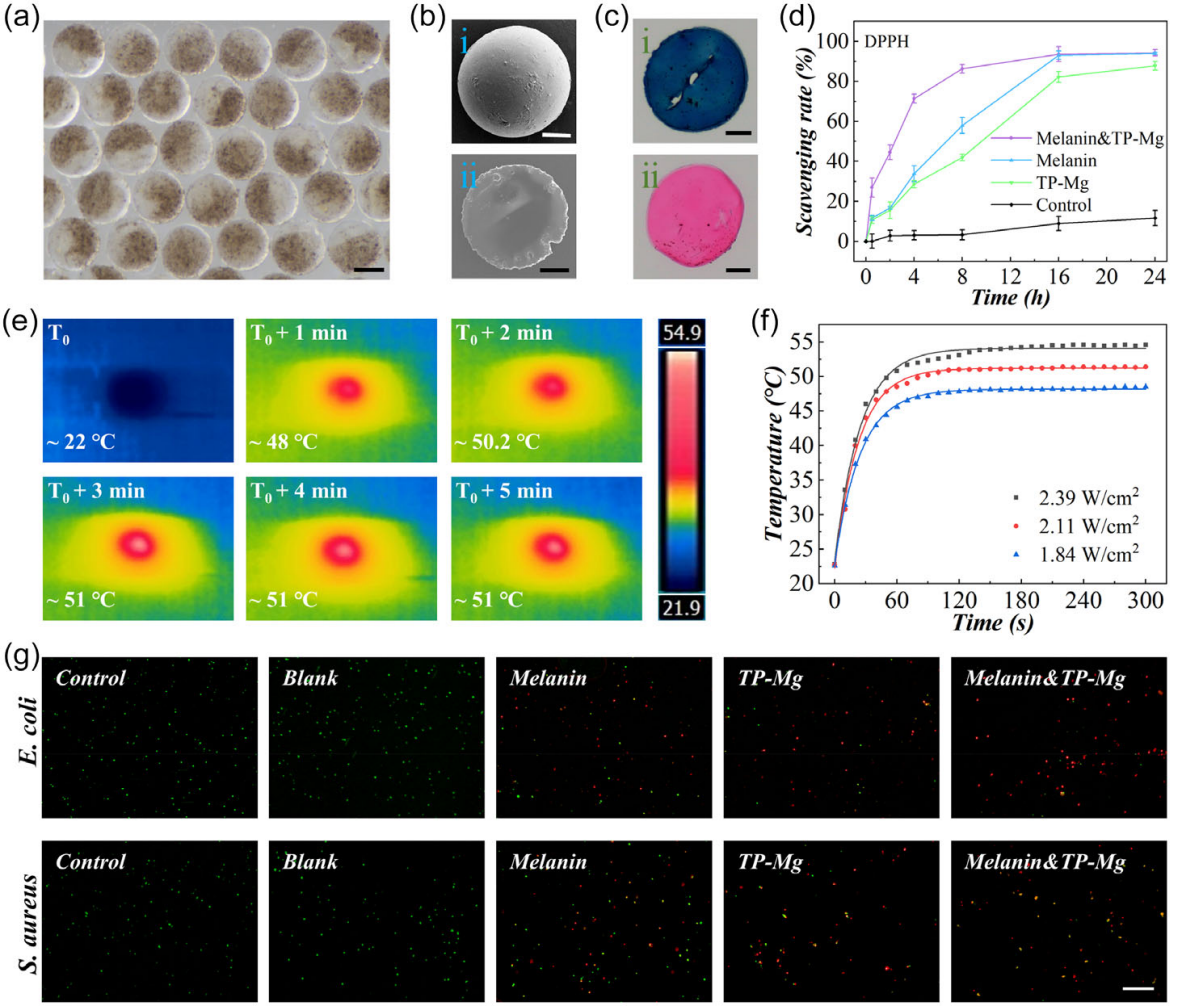
Multifunction evaluation of Janus microparticles. a) Optical image of Janus hydrogel microparticles. b) The SEM images of a Janus microparticle (i) and its slice (ii). c) The optical images of microparticle slice stained by Masson (i) and H&E (ii) dyes. d) The DPPH‐scavenging activity of different microparticles. e) The thermal images of microparticles layer under NIR irradiation. f) The recorded temperature change of microparticles under different NIR powers. g) The Live (green)‐dead (red) staining of bacteria received different treatments. Scale bars are 200 μm in (a), 100 μm in (b–c), and 50 μm in (g). Sample size *n* = 3 for all experiments.

After fully exposed under UV light, the final hydrogel microparticles were obtained. The nanoparticles can be selectively integrated into the hemisphere of microparticles by adding into the precursor, obtaining Janus microparticles with different components, including melanin Janus microparticles (one hemisphere with melanin nanoparticles), TP‐Mg Janus microparticles (one hemisphere with TP‐Mg nanoparticles), and melanin&TP‐Mg Janus microparticles (one hemisphere with melanin nanoparticles and the other one with TP‐Mg nanoparticles). Janus structure made them separated in the dosage process, avoiding the potential side reactions. The final Janus microparticles presented spherical shape with uniform tight structure on the surface and inside, as observed by optical microscope and scanning electron microscopy (SEM) (**Figure** **3**a,b). In addition, the Janus microparticles retained the structure property of collagen, resembling to ECM, as proved by the staining of Masson dye and hematoxylin eosin (H&E) (Figure 3c).

The melanin&TP‐Mg Janus microparticles were imparted with the curative effect of both melanin and TP‐Mg nanoparticles after integration, verified by the following tests. The antioxidant property of Janus microparticles was assessed by reaction with DPPH. Compared to Janus microparticles integrated with only one kind of nanoparticles, the melanin&TP‐Mg Janus microparticles presented faster elimination rate of DPPH (Figure 3d). This synergetic antioxidant effect can be attributed to combination of the nanoenzyme activities of melanin nanoparticles and free radical elimination of phenolic hydroxyl groups of TP‐Mg nanoparticles. The photothermal effect of melanin&TP‐Mg Janus microparticles was then studied. It is observed that the local temperature of microparticles dramatically increased in the first minute, slowly increased until reached the highest temperature in the next two minutes, and then kept stable, keeping the same under all the tested NIR powers (Figure 3e,f). The highest temperatures were near 48, 51, and 54 °C at the power of 1.84, 2.11, and 2.39 W cm^−2^, respectively. 2.11 W cm^−2^ was selected for following tests for it provided suitable heating behavior, while might not cause severe discomfortableness and hurt. The cycle test further proved its stable heating activity (Figure S9a, Supporting Information).

Antibacterial capacity is essential to the healing of diabetic wounds for they are easy to be infected due to the weak immune protection. To study the antibacterial effect of Janus microparticles, *Staphylococcus aureus* (*S. aureus*) and *Escherichia coli* (*E. coli*) were chosen as the bacteria models. The bacteria suspension was added with blank hydrogel microparticles (Blank group), melanin Janus microparticles (Melanin group), TP‐Mg Janus microparticles (TP‐Mg group), and melanin&TP‐Mg Janus microparticles (Melanin&TP‐Mg group). The bacteria that received no extra treatments were set as Control group. Melanin group and Melanin&TP‐Mg group received extra NIR irradiation. NIR‐induced heating effect caused death of bacteria via heating hurt in the melanin group, but the final rate was not satisfactory. TP‐Mg could destroy the biofilm system and induce the intracellular ROS generation caused by Mg—O chemical structure and phenolic hydroxyl group to achieve sterilization.^[^ ^19^ ^]^ Compared to the melanin group and TP‐Mg group, treatment of Melanin&TP‐Mg Janus microparticle realized better sterilization (Figure 3g and S8b‐d, Supporting Information), which was benefited from the combination of NIR‐induced heating hurt and TP‐Mg‐assisted chemical damage. These results revealed the efficacy of Melanin&TP‐Mg Janus microparticles with multiple strategies for synergetic antioxidant and antibacterial. Based on those findings, Janus microparticles integrated with both melanin and TP‐Mg nanoparticles were chosen for following tests.

**Figure 4:**
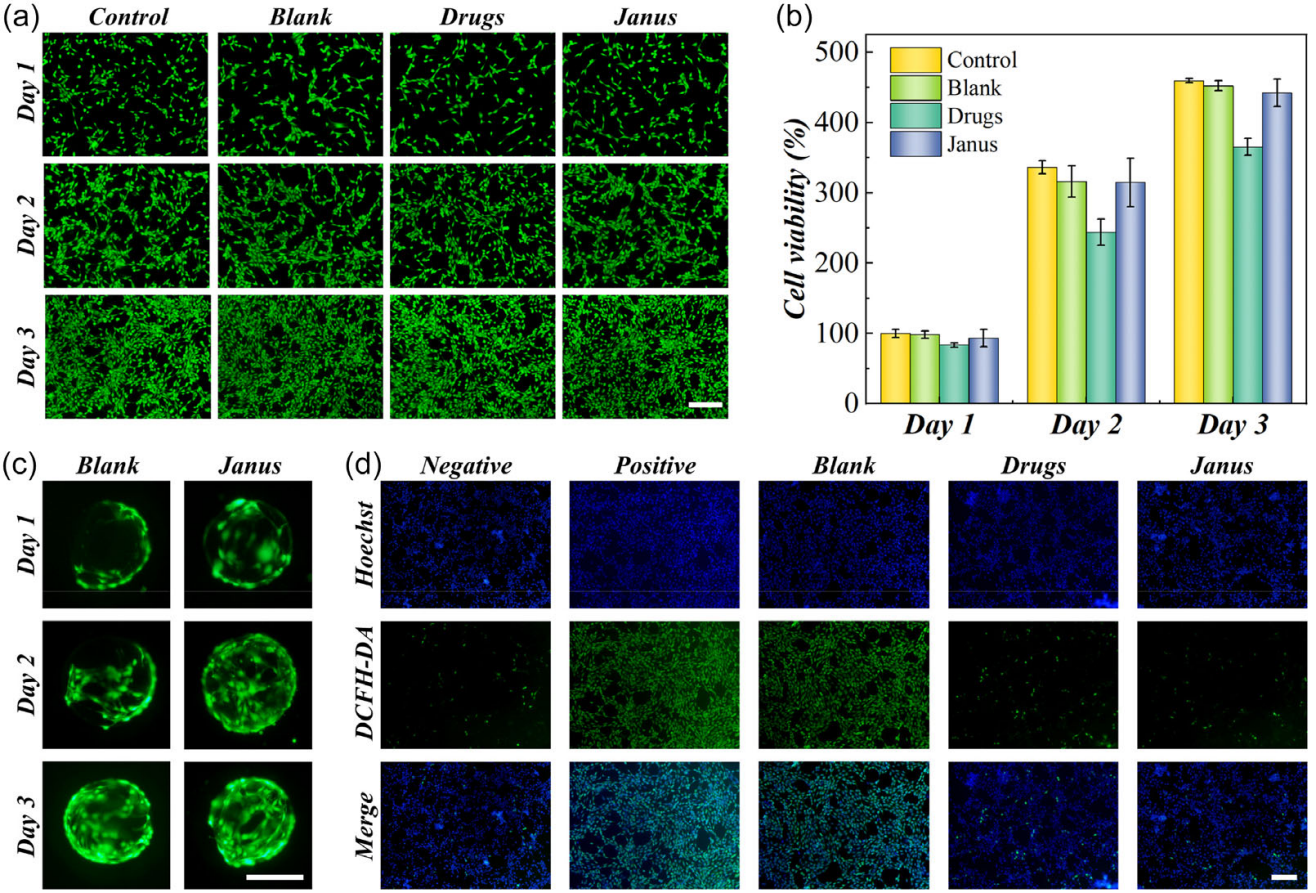
In vitro biocompatibility and ROS scavenging capacity of Janus microparticles. a) The fluorescent images of cells in different groups over 3 days. b) The statistics of cell viability during 3 days. c) The fluorescent images of cells adhered to the surface of hydrogel microparticles and Janus microparticles. d) The ROS staining of H_2_O_2_‐treated cells after receiving different treatments. Scale bars are 200 μm in (a) and (c–d). Sample size *n* = 6 for all experiments.

To verify the application potential of Janus microparticles in promoting wound healing, first, their curative effect was verified in vitro. The biocompatibility of nanoparticles and microparticles was assessed by co‐incubation with erythrocytes and NIH 3T3 cells. When incubated with erythrocytes, all the nanoparticles and hydrogel microparticles showed low influence to erythrocytes (Figure S10, Supporting Information). The results of Calcein‐AM‐stained living cell and cell viability measured by cell counting kit‐8 (CCK‐8) assay revealed the negligible influence of the hydrogel microparticles with nanoparticles to the healthy morphology and normal metabolization of 3T3 cells (**Figure** **4**a,b). In contrast, the proliferation rate of cells treated by free nanoparticles was slower than the hydrogel microparticles. More interestingly, the cells can adhere to the surface of hydrogel microparticles and proliferate along the 3 days’ culture (Figure 3c). In addition, when the cells were cultured under hydrogen peroxide (H_2_O_2_) environment, the utilization of both free nanoparticles and Janus microparticles can decrease the ROS content to relief oxidant stress (Figure 4d). These results indicated that Janus microparticles achieved almost the same antioxidant effect of nanoparticles to cells and further reduced the influence of nanoparticles to cell proliferation.

**Figure 5:**
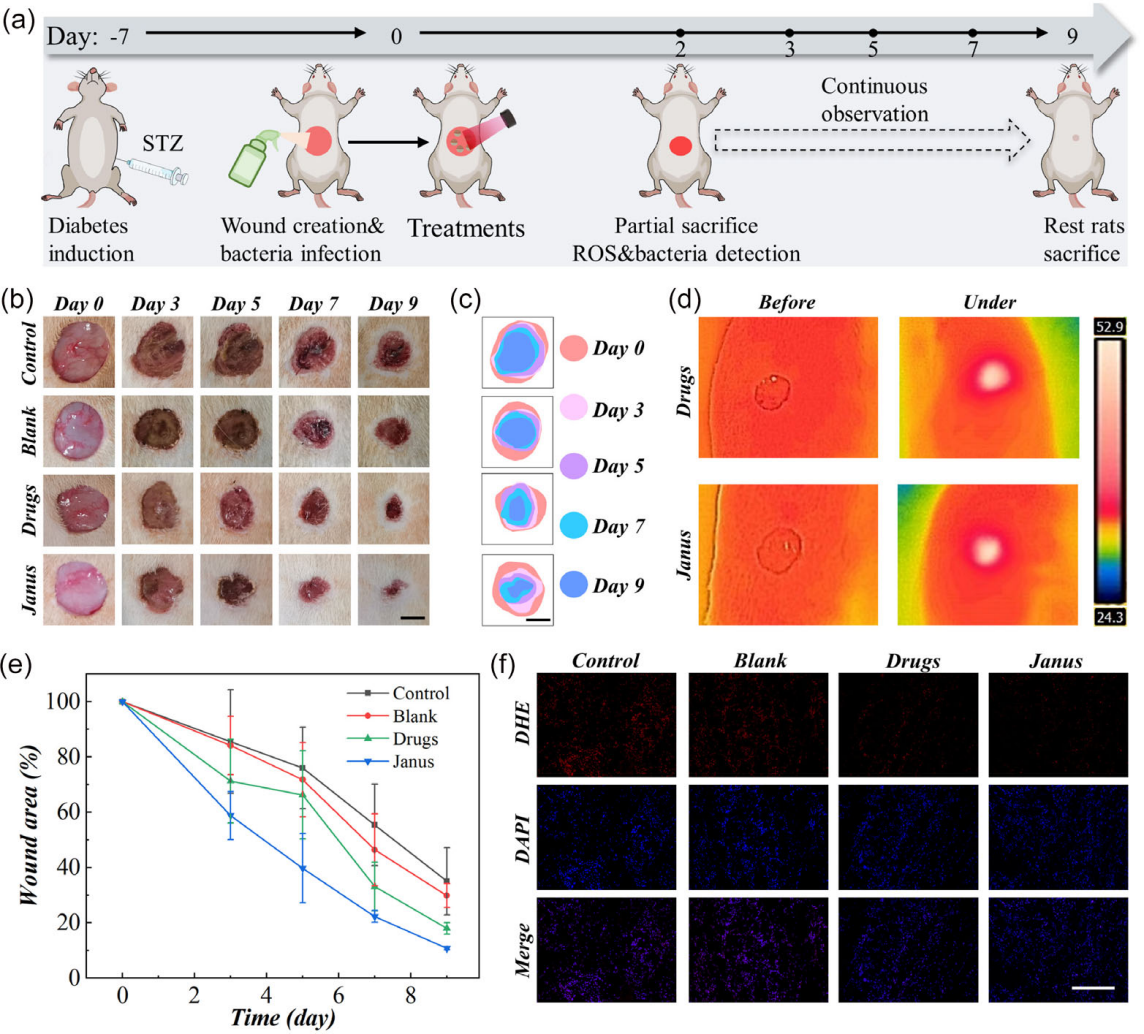
In vivo evaluation of the antibacterial and ROS scavenging capacity of Janus microparticles. a) The schematic illustration of treatments on rats. b) The recorded digital images of wounds in four groups. c) The overlapped area of wounds at different recording point. d) The thermal images of wounds treated by (i) nanoparticles suspension and (ii) Janus microparticles before and under NIR irradiation. e) The statistical healing process of wound area. f) The fluorescence images of ROS staining in wounds, stained by DHE. Scale bars are 0.5 cm in (b–c) and 200 μm in (f). Sample size *n* = 8.

The healing of diabetic wound is hindered by excess ROS, inflammation, and abnormal ECM remolding caused by matrix metalloproteinase‐9 (MMP‐9) secretion. To examine the healing effect of Janus microparticles to address those conditions in practical, animal test was then carried out (**Figure** **5**a). Streptozotocin (STZ) was used to induce the diabetes of Sprague‐Dawley (SD) rats by intraperitoneal injection. The blood glucose and body weight were detected to verify the successful modeling of diabetes (Figure S11a‐b, Supporting Information). The bacteria‐infected wounds were created on these diabetic rats by removing the full‐thickness skin at the diameter of near 1 cm on the back and sprayed with 100 μL bacteria suspension. Those rats were then divided into four groups, encompassing Blank group received blank hydrogel microparticles treatment, Drugs group received treatment of melanin nanoparticles and TP‐Mg nanoparticles suspension, Janus group received Janus hydrogel microparticles treatment, and Control group received phosphate buffer saline (PBS) treatment. After therapeutics treatments, the wounds in latter two experimental groups were radiated by NIR laser, and the thermal images of wounds before and under NIR irradiation were recorded (Figure 5d). The results demonstrated that the photothermal heating behavior could be achieved on rats as well. The photos of wounds at day 0, 3, 5, 7, and 9 were recorded to analyze the difference of healing process in different groups (Figure 5b,c). At day 2, the status of bacterial infection and ROS content was examined. The bacteria on the surface of wounds were collected and cultured on the Luria‐Bertani (LB) agar plate. The results indicated that the treatment of free nanoparticles and Janus microparticles significantly reduced the number of bacteria, compared to the Control group (Figure S11c, Supporting Information). ROS content was qualitatively analyzed by dihydroethidium (DHE) staining. In Drugs group and Janus microparticles group, the observed ROS were less than the Control group and Blank group as well (Figure 5f). These results revealed that the existence of melanin and TP‐Mg nanoparticles could kill the bacteria and decrease ROS in the diabetic wounds.

**Figure 6:**
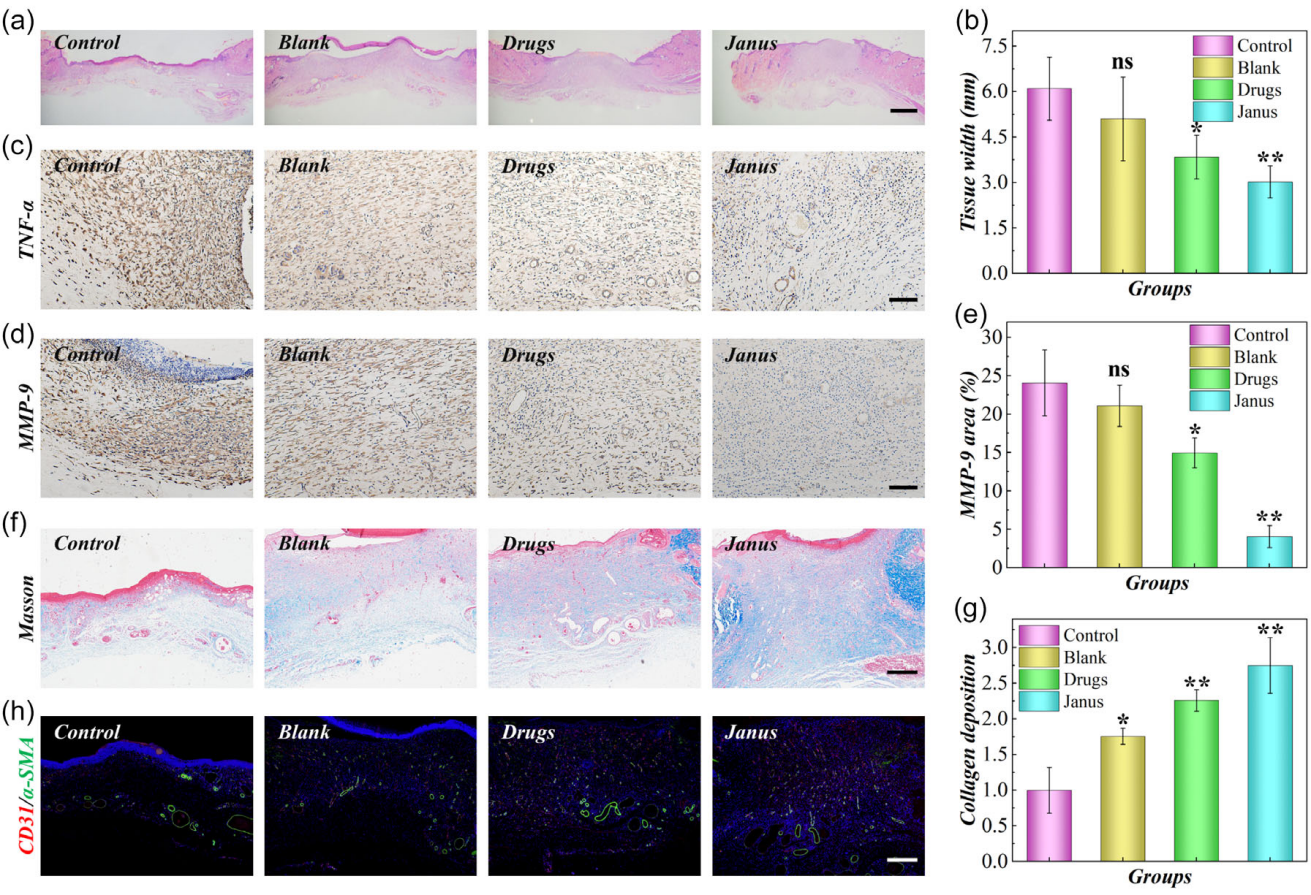
Histochemical staining results. a) The H&E staining images of tissues in different groups. b) The statistics of tissue width in different group. c,d) The IHC staining images of TNF‐α (c) and MMP‐9 (d). e) The diagram of MMP‐9 positive area. f) The Masson staining images of tissues in different group. g) The normalized collagen‐deposited area in different groups. h) The immunofluorescent staining images of α‐SMA and CD31. Scale bars are 1 mm in (a), 100 μm in (c–d), 400 μm in (f) and (h). Sample size *n* = 8 for all experiments. ns: not significant, *p < 0.05, **p < 0.01.

At day 9, the wounds in Janus group were almost closed, followed by the Drugs group (Figure 5e). In contrast, the wound area in Control group and Blank group was much larger. The regenerated tissues in all the groups were harvested to be stained to assess the healing state in detail. As presented by H&E staining, the thickness of wounds in Janus groups was thicker and more narrow than other three groups, which was normally regarded as better wound healing performance, followed by the Drugs group, Blank group, and Control group in turn (**Figure** **6**a,b and S11d, Supporting Information). The inflammatory status of the wounds was evaluated by the immunohistochemical (IHC) staining of tumor necrosis factor‐α (TNF‐α). It can be found that Janus group secreted the lowest inflammatory factor, proving relived inflammatory environment (Figure 6c). In contrast, the wounds in Drugs group secreted more inflammatory factor but also lower than the Blank group and Control group. Additionally, over expression of MMP‐9 was widely observed in diabetic wound due to increased ROS, which is harmful to wound healing. For those groups treated with nanoparticles and Janus microparticles, the level of MMP‐9 was also significantly reduced compared to others (Figure 6d,e).

The remodeling of wounds was important stage of wound healing, along with collagen deposition and revascularization. To investigate the remodeling level of wounds in all groups, Masson staining for collagen and staining of CD31 and α‐SMA for vascular structure were carried out. The wounds in Janus groups were found with more collagen than the Control group due to less secretion of MMP‐9. It is worth noting that the use of hydrogel microparticles also enhanced the deposition of collagen, which may be attributed that the microparticles with ECM‐like components could play as the scaffold to promote ECM recovery (Figure 6f, g). Revascularization is important for nutrient transport and exchange to accelerate wound healing. The staining results of CD31 and α‐SMA demonstrated that the vessel density of Janus group was the highest. Compared to Control group, the revascularization level of Drugs group and Blank group was also better (Figure 6h and S12, Supporting Information). In general, these findings supported the practical value of Janus microparticles for healing of diabetic wounds.

## 3 Conclusion

In summary, Janus microparticles incorporated with melanin nanoparticles and TP‐Mg nanoparticles were successfully manufactured by microfluidics for diabetic wounds treatment. Both melanin nanoparticles extracted from black hair and fabricated TP‐Mg nanoparticles have exhibited great free radical‐scavenging capacity and strong sterilization activity, but at different mechanisms. The manufactured hydrogel microparticles retained the components of collagen resembling to ECM, which was beneficial for cell adherence and proliferation. After integration of these two nanoparticles into Janus microparticles by microfluidics, the resultant Janus microparticles were imparted with the outstanding capacities of these nanoparticles, including antioxidant and antibacterial. When applied to the administration of diabetic wound, they achieved ROS reduction and bacteria killing in the early healing stage. Additionally, they could reduce inflammation and MMP‐9 secretion to accelerate tissue remolding. Therefore, it is believed that our Janus hydrogel microparticles with integration of melanin and TP‐Mg nanoparticles can administrate and combine the curative effect of these nanoparticles to achieve better healing. It should be noted that this work just focused on providing a conception proof of antioxidant and antibacterial Janus microparticles with melanin and TP‐Mg nanoparticles. It lacked the verification investigation of multispecies medicinal treatment. In addition, their specific mechanism and the involved signaling pathways within the body needed to be further clarified in the subsequent work.

## 4 Experimental Section

4.1

4.1.1

##### Materials

MgSO_4_·7H_2_O was supplied by RHAWN (China), and TP was provided by Shanghai Yuanye Bio‐Technology (China). Nanjing Jiancheng Bioengineering Institute (China) provided hydroxyl‐free radical assay kit. Recombinant human II collagen was purchased from Jiangsu JL and Biotech Co., Ltd (China). Propidium iodide (PI), SYTO, and NaOH were supplied by Macklin (China). Hair was devoted by the author. 2‐hydroxy‐2‐methylpropiophenone (HMPP) was bought from Sigma‐Aldrich. Crystal violet solution was obtained from Solarbio (China). Calcein‐AM, CCK‐8 Assay, and Hoechst 33342 were supplied by Beyotime (China). All the used staining dyes, antibodies, and DCFH‐DA were obtained from Servicebio (China). DPPH and PTIO were supplied by Meryer (China). NIH 3T3 cells were provided by The Cell Bank of the Chinese Academy of Sciences (China). The 8‐12‐week‐old male Sprague‐Dawley rats were provided by Drum Tower Hospital. Animal Investigation Ethics Committee of Southeast University has examined and approved all animal experimental programs (20250115001).

##### TP‐Mg Synthesis

23 mL PBS was used to dissolve 50 mmol TP and was added with 1 mL Mg^2+^ solution with different Mg^2+^ molar contents, heated at 45 °C. After one hour, 1 mL NaOH solution at different concentration was added, reacting for 8 h. The final suspension was centrifuged at 6000 rpm × 30 min. The supernate was transferred into ultrafiltration tube (MWCO 10 kDa) for cleaning by PBS. If there was sediment, the sediment was continuously cleaned by repeated centrifuging at 6000 rpm × 30 min and suspending. After that, the obtained nanoparticles suspension was washed by ddH_2_O in ultrafiltration tube for characterization and weight after frozen‐drying.

##### Bacteria Proliferation Inhibition

Bacteria at the concentration of 10^6^ CFU mL^−1^ were cultured in LB media with TP‐Mg nanoparticles (100 μg mL^−1^) that were fabricated from different parameters. After 6 h, the bacteria suspension was centrifuged to collect the bacteria and then suspended in 200 μL PBS, which was added into a 96‐well plate to record the absorbance value at 600 nm under microplate reader.

##### Biofilm Test

For biofilm destruction test, 150 μL *S. aureus* bacteria suspension at 10^5^ CFU mL^−1^ in LB media was incubated in 48‐well plate at 37 °C for 24 h, and then the old media was removed and the formed mature biofilm was washed by PBS. TP‐Mg suspension at 20, 40, 60, 80, and 100 μg mL^−1^ was added, following 12 h incubation. Subsequently, the suspension was removed and the biofilm was washed by PBS, fixed by methanol for 15 min, and dried. The biofilm in the wells was stained by 200 μL 0.1% crystal violet solution for 30 min. After that, PBS was used to wash the wells for 5 times, following drying at 37 °C. 220 μL absolute ethyl alcohol was added to dissolve the crystal violet, and then 200 μL of that was suck out for imaging and measuring absorbance at 570 nm. For formation inhibit of biofilm, 10^5^ CFU mL^−1^ suspension at the volume of 150 μL was cultured in a 48‐well plate. The final LB culture media contained 4, 8, 12, 16, and 20 μg mL^−1^ TP‐Mg nanoparticles, respectively. After one day culture at 37 °C, the bacteria suspension was suck out and the wells were cleaned by PBS. Following staining of crystal violet was at the same way with biofilm destruction test.

##### Extraction of Melanin Nanoparticles

Per 0.5 g of black hair was immersed into 10 mL NaOH solution for reaction at 60 °C. The tested reaction time was from 2 to 12 h at the condition of 1 M NaOH. The tested NaOH concentration was from 3 to 13 M at the condition of 12 h reaction time. After that, the obtained suspension was centrifuged at 5000 rpm to collect the sediment and then suspended by ddH_2_O for cleaning, repeated for five times to harvest the final nanoparticles.

##### ColMA Synthesis

Per 1 g collagen was dissolved in 10 mL PBS to obtain 10% solution, which was then added with 0.5 mL MA under stirring. The pH value was adjusted and kept between 8–9 by 1 M NaHCO_3_ during 1 h reacting. Then, the mixture was centrifuged and transformed into 14 kDa dialysis tube for dialysis, lasting 5 days. The final obtained solution was frozen‐dried after its pH value was changed to near 7.2 by 1 M NaHCO_3_.

##### Microfluidics Device Construction

The microfluidic device was assembled by several capillaries. A θ tube was pushed over after heating and then polished to the inner diameter of near 200 μm. A square tube at the inner side of 1.1 mm was used as the observation tube. A capillary at the inner diameter of 800 μm was coaxially arranged with θ tube within the channel of square tube. All the tubes were fixed on a glass slide, and all the joints were cured by AB glue.

##### Janus Microparticles Fabrication

20% ColMA solution suspended with melanin nanoparticles and TP‐Mg nanoparticles was pulled into the channel of microfluidic device by injection pump as continuous phase. HMPP as photo‐initiator was also added into ColMA solution. Plant oil was used as dispersed phase to shear the continuous phase at the nozzle of θ tube, forming dispersed microdroplets in the collection tube. The droplets were pre‐polymerized into Janus hydrogel particles under UV point light. The collected particles were further placed under UV laser irradiation for 10 min for fully polymerization. Alcohol was used to clean the resultant microparticles and replaced by ddH_2_O.

##### Slicing and Staining of Microparticles

Microparticles were immersed into 15% sucrose solution, 30% sucrose solution, and optimal cutting temperature compound in turn, following freezing and slicing at the thickness of 40 μm. The staining scheme of H&E staining and Masson staining was consistent to traditional methods.

##### Characterization

The optical image was captured by a CCD camera‐equipped optical microscope. TEM (JEM‐2100, JEOL) and field emission SEM (Ultra Plus, Zeiss) were used to observe the shape of nanoparticles and microparticles. UV–vis spectrum was obtained from UV–vis spectrophotometer (UV‐2700i, SHIMADZU). XPS spectrum data were measured under XPS (Thermo Scientific K‐Alpha). FTIR spectrum was obtained from FTIR spectrophotometer (Nicolet IS50, ThermoFisher Scientific). Hydrodynamic particle size was measured by using DLS particle sizer (Zetasizer lab, Malvern). The absorbance was recorded under microplate reader (Infinite E Plex, TECAN).

##### Antioxidant Test

The ·OH scavenging capacity of nanoparticles was quantified by using hydroxyl free radical assay kit according to the instruction. DPPH working solution at the absorbance of round 1.0 at 517 nm was prepared by dissolving DPPH into absolute ethyl alcohol. Melanin nanoparticles were centrifuged and suspended to final 0.1, 0.2, 0.3, 0.4, and 0.5 mg mL^−1^ by DPPH working solution. After 30 min incubation, these solutions were centrifuged to obtain the supernate for absorbance measurement at 517 nm. PTIO was dissolved in the solution at the pH value of 4, 7, and 9, respectively, at the concentration of 0.15 mg mL^−1^. After incubation with melanin nanoparticles for 6 h at 37 °C, these solutions were centrifuged to obtain the supernate for absorbance measurement at 557 nm. The test protocol of TP‐Mg nanoparticles kept the same with the melanin nanoparticles. As for test of microparticles, melanin group contained Janus microparticles from 0.5 mL melanin/ColMA solution and 0.5 mL pure ColMA solution. TP‐Mg group contained Janus microparticles from 0.5 mL TP‐Mg/ColMA solution and 0.5 mL pure ColMA solution. Melanin&TP‐Mg group contained Janus microparticles from 0.5 mL melanin/ColMA solution and 0.5 mL TP‐Mg/ColMA. All of them were frozen‐dried and then directly immersed into the DPPH working solution. After 30 min incubation, these solutions were centrifuged to obtain the supernate for absorbance measurement at 517 nm. The eliminating efficiency was quantified by adopting the same way of nanoparticles.

##### Photothermal Performance

For test of melanin nanoparticles suspension, 200 μL melanin suspension at different concentration was added into a square model at 1 × 1 cm. NIR laser (808 nm) vertically irradiated the suspension, and a handle thermal imaging was used to record the surface temperature per 10 s. For test of Janus microparticles, they were arranged into single layer at the area of 1 × 1 cm in a glass slide. NIR lasers at the power of 1.84, 2.11, and 2.39 W cm^−2^ were used to irradiate the covered area, and the change of local temperature was recorded. Cyclic test was carried out at the power of 2.11 W cm^−2^, where the laser light was turned on in the first 3 min and turned off in the latter 3 min in every cycle.

##### Antibacterial Test

*S. aureus* and *E. coli* were resuscitated and incubated in LB liquid media. After centrifugation, the bacteria were suspended in PBS, reaching 0.5 MCF (namely, 1.5 × 10^8^ CFU mL^−1^). The obtained bacteria suspension was cultured with nothing, blank hydrogel microparticles, TP‐Mg Janus microparticles, melanin Janus microparticles, and melanin&TP‐Mg Janus microparticles, respectively. For latter two groups, the suspension was irradiated with NIR per 12 h. After 24 h incubation at 37 °C, half of the suspension of bacteria was used for staining of PI and SYTO for observation under fluorescence microscope. The last half of the suspension was diluted 10^3^ times. 50 μL diluted suspension was spread in LB agar plate. After 24 h incubation at 37 °C, the formation of bacterial colony was recorded.

##### Cell Test

3T3 cells were incubated with blank microparticles, nanoparticles, and Janus microparticles as experimental groups while that in pure culture media as control group. Among 3 days, CCK‐8 assay was employed to measure the cell viability every day, and the morphology of cells in the wells and on the surface of microparticles was checked after Calcein‐AM staining. For oxidant stress reliving test, 3T3 cells were cultured with incomplete culture media with 100 μM H_2_O_2_ to induce the oxidant stress of cells. Hydrogel microparticles, nanoparticles and Janus microparticles were incubated with these cells, respectively. Another group of cells treated with no H_2_O_2_ was set as negative control. After 24 h, the old culture media were suck out and the cells were washed using PBS, following staining of Hoechst 33342 and DCFH‐DA. The fluorescent images of stained cells were imaged under Invert fluorescent microscopy. Fresh erythrocytes were isolated from the blood of a rat and suspended in PBS for hemolysis test. Erythrocyte suspensions were added with PBS as negative control, deionized water as positive control, nanoparticles suspension as drugs group, and Janus microparticles as Janus group, respectively. Free nanoparticles suspension was set as detection control for drugs group. After 24 hour standing still, they were imaged and their supernates were suck out for absorbance measurement at 545 nm.

##### Animal Test

Every SD rat was injected with streptozotocin at the dosage of 70 mg per 1000 g of rat weight to induce the diabetes. The blood glucose level of rats before injection and at 48th and 72nd h after injection were detected while the body weight of rats during one week were measured. After one week, all the rats had wounds at the diameter of near 1 cm on their back and infected by 100 μL 0.5 MCF bacteria suspension of *E. coli* and *S. aureus*. They were then randomly separated into four groups, encompassing Blank group received therapeutics of hydrogel microparticles, Drugs group received therapeutics of melanin and TP‐Mg nanoparticles following NIR irradiation, Janus group received therapeutics of Janus microparticles following NIR irradiation, and Control group treated with PBS. At day 0, the thermal images of local wounds were recorded before and under NIR irradiation. At day 2, 100 μL PBS was used to clean the wounds and collected for infection evaluation. After that, 1/3 rats were killed to harvest the wounds for frozen section. The healing process of other wounds was recorded at day 3, 5, 7, 9. At day 9, all the wounds were harvested for paraffin section.

##### Histological Evaluation

These wounds from day 2 were used for ROS staining by DHE. The wounds from day 9 were sliced after paraffin embedding for the other staining, including H&E staining, IHC staining of TNF‐α and MMP‐9, Masson staining, immunofluorescent staining of α‐SMA and CD31.

##### Statistics Analysis

All the count data were calculated using Excel and represented as mean ± standard derivation. *n* ≥ 3. The data processing of histological staining employed ImageJ. Significance analysis used Student's *t*‐test by SPSS software, and the difference is significant only when *P* < 0.05.

## Supporting Information

Supporting Information is available from the Wiley Online Library or from the author.

## Conflict of Interest

The authors declare no conflict of interest.

## Supporting information

Supplementary Material

## Data Availability

The data that support the findings of this study are available from the corresponding author upon reasonable request.
