# Supplementary material for: Janus Hydrogel Microparticles with Tea Polyphenol/Melanin Nanoparticles Integration from Microfluidics for Wound Healing
Source: Small Sci. 2025 Aug 27;5(11):2500322. doi: 10.1002/smsc.202500322 (PMC12622484; doi:10.1002/smsc.202500322)
Supplement: Supplementary file 1 — Supplementary Material [file SMSC-5-2500322-s001.pdf]

Supporting Information

Janus Hydrogel Microparticles with Tea Polyphenol/Melanin Nanoparticles Integration from Microfluidics for Wound Healing

*Zhiqiang Luo, Danqing Huang, and Yuanjin Zhao**

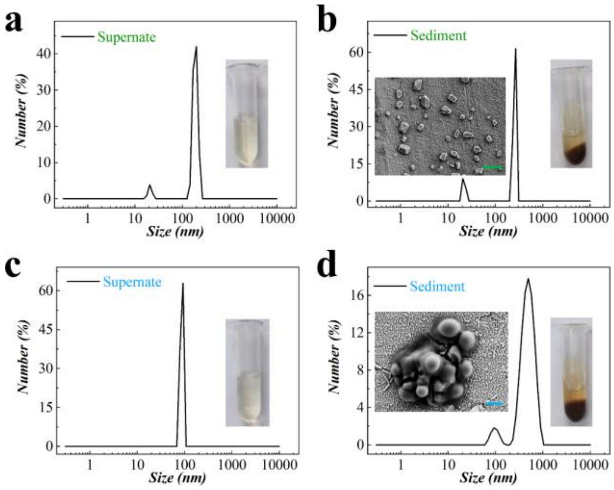

Figure S1. Characterization of collected supernates and sediments after centrifugation. (a-b) The hydrodynamic size of TP-Mg nanoparticles in the (a) supernate and (b) sediment from the parameter of 0.05 mmol Mg^{2+}/5 M NaOH and its SEM image. (c-d) The hydrodynamic size of TP-Mg nanoparticles in the (c) supernate and (d) sediment from the parameter of 0.1 mmol Mg^{2+}/0.5 M NaOH and its SEM image. Scale bars are 1 μm in (b) and 0.5 μm in (d).

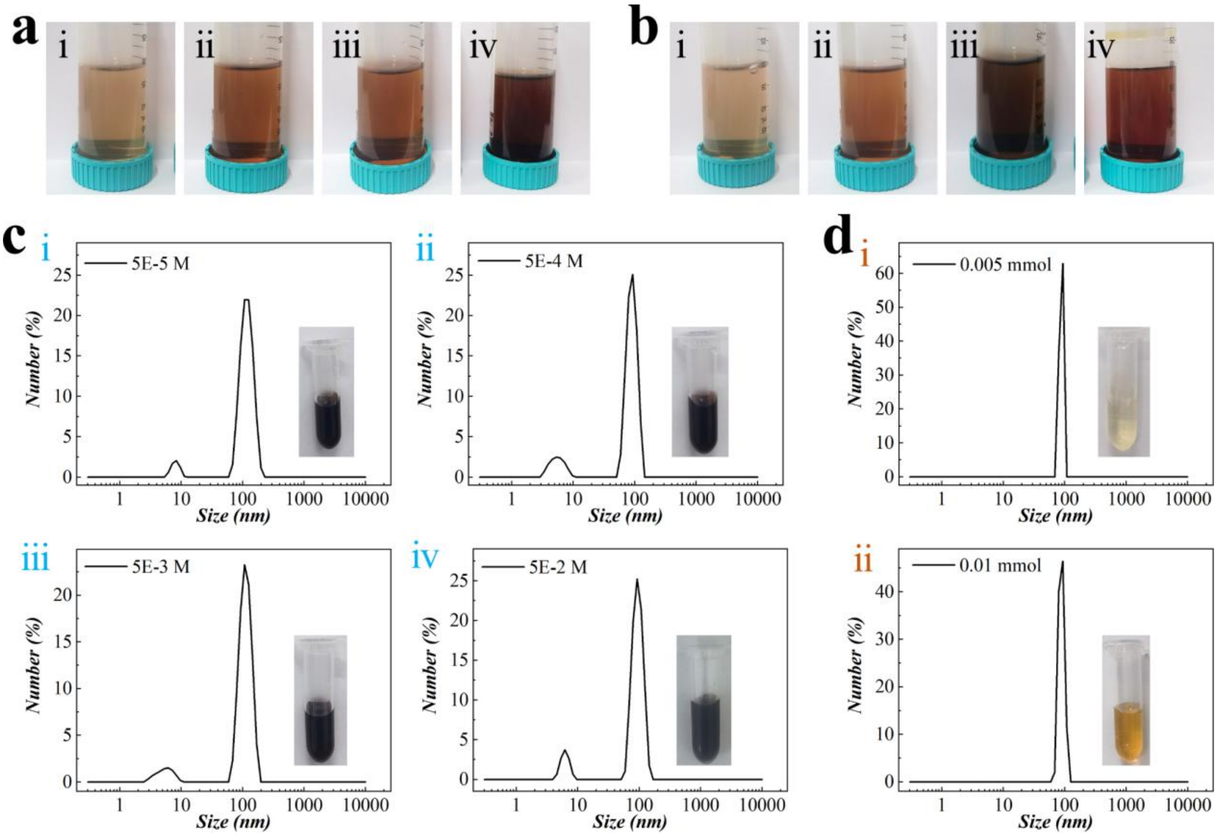

Figure S2. Fabrication optimization of TP-related nanoparticles. (a-b) The imaged images during fabrication process from the parameter of (a) 0.05 mmol Mg^{2+}/0.05 M NaOH and (b) 0.05 mmol Mg^{2+}/0.5 M NaOH at the timepoint of (i) initial 0 h, (ii) 1 h before NaOH addition, (iii) 1 h after NaOH addition and (iv) final 9 h. (c) The influence of NaOH concentration to the hydrodynamic size of TP-Mg nanoparticles. (d) The influence of Mg content to the size of TP-Mg nanoparticles. The inserted images in (c-d) were the finally obtained suspension products.

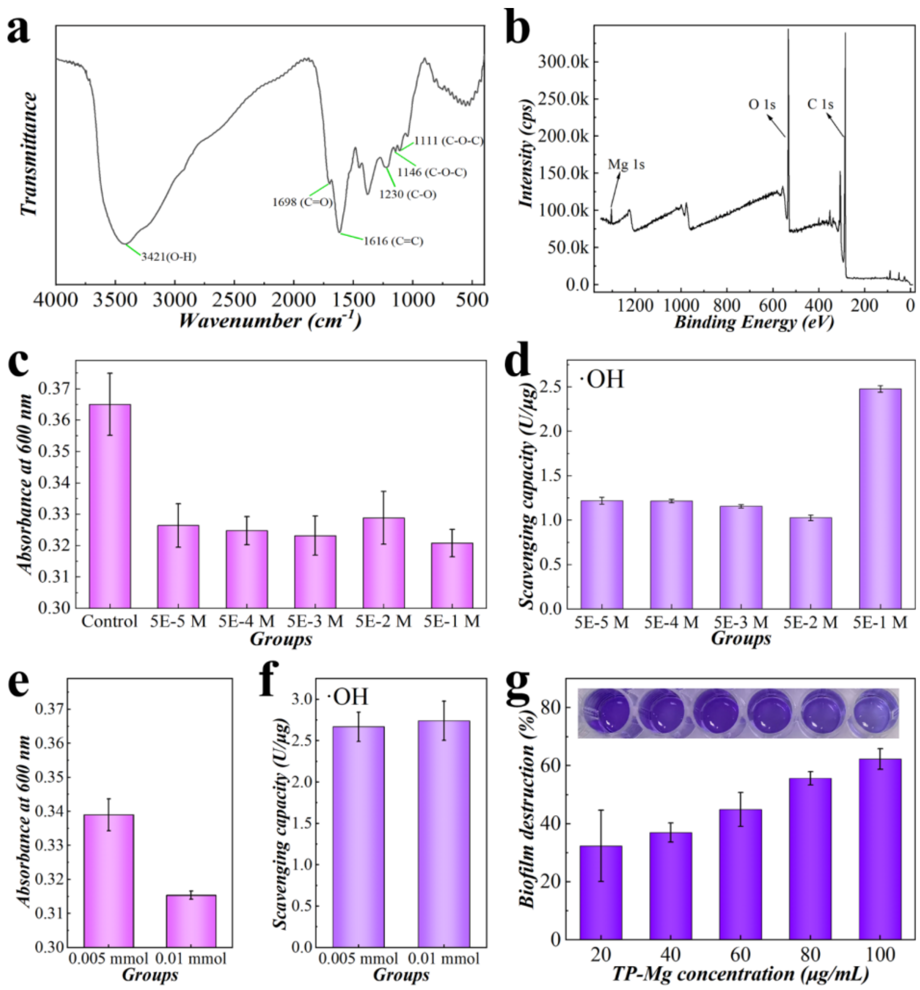

Figure S3. Characterization of TP-Mg nanoparticles. (a) The FTIR spectrum of nanoparticles fabricated from the parameter of 0.05 mmol Mg^{2+}/0.05 M NaOH. (b) The XPS spectrum of TP-Mg nanoparticles. (c) The inhibition capacity of nanoparticles fabricated from different NaOH concentration to bacteria proliferation. (d) The ·OH scavenging capacity of nanoparticles fabricated from different NaOH concentration. (e) The inhibition capacity of nanoparticles fabricated from different Mg^{2+} content to bacteria proliferation. (f) The ·OH scavenging capacity of nanoparticles fabricated from different Mg^{2+} content. (g) Anti-biofilm capacity of TP-Mg nanoparticles. Sample size $n = 3$ for all experiments.

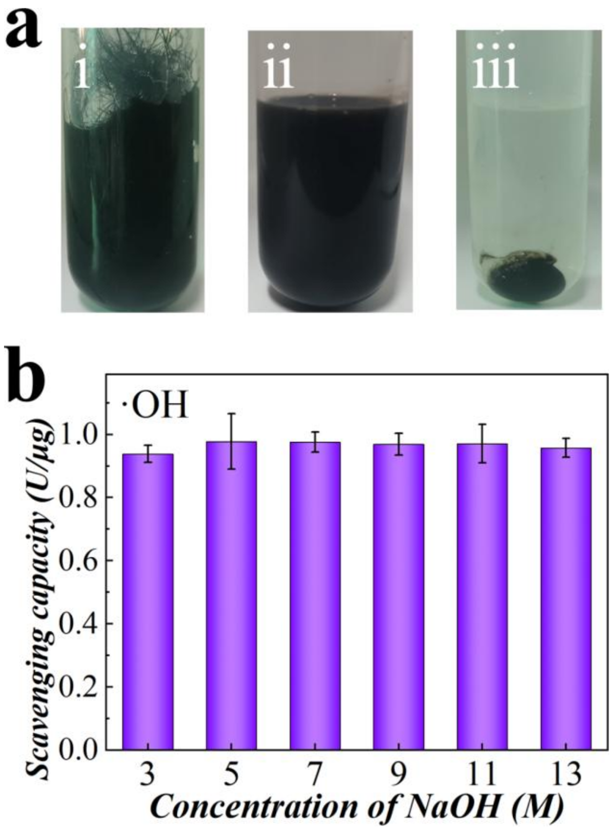

Figure S4. Fabrication of melanin nanoparticles. (a) The optimal images of mixture at the timepoint of (i) initial 0 h, (ii) 12 h and (iii) obtained sediment after cleaning. (b) The ·OH scavenging capacity of melanin nanoparticles fabricated from different NaOH concentration. Sample size $n = 3$.

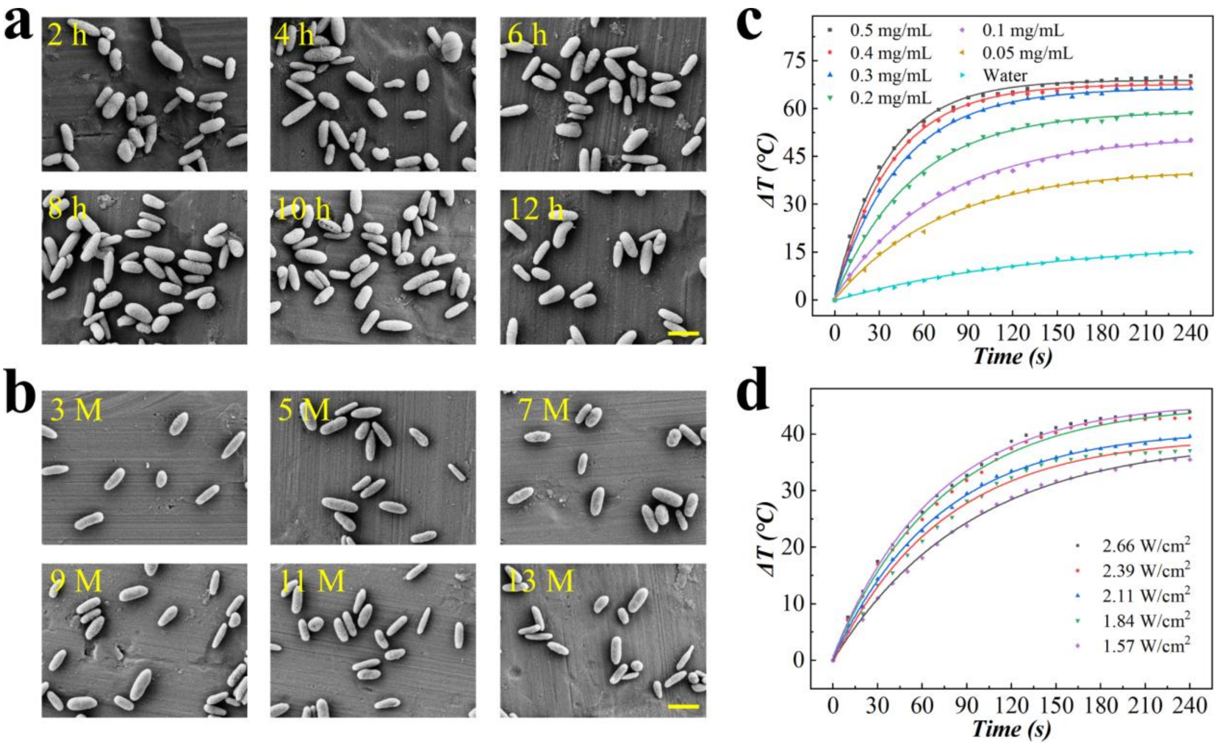

Figure S5. Characterization of melanin nanoparticles. (a-b) The influence of (a) reaction time and (b) NaOH concentration to the morphology of melanin nanoparticles. (c-d) The alternative of local temperature of melanin suspension with different concentration(c) and under different NIR power (d). ΔT: temperature variation. Scale bars are 1 μm in (a-b).

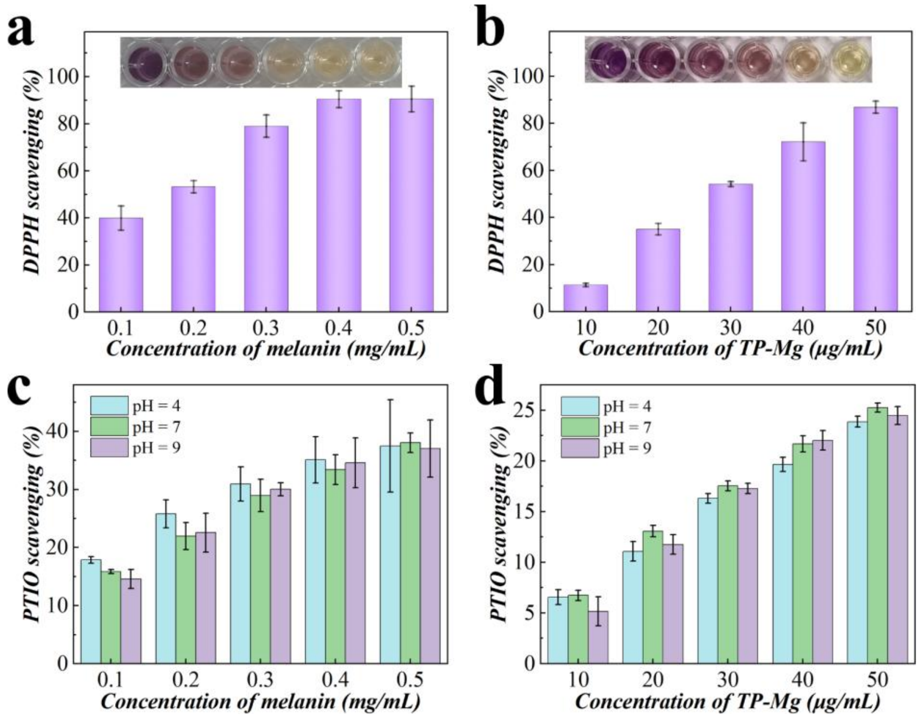

Figure S6. Antioxidant evaluation of fabricated nanoparticles. (a) The DPPH scavenging rate of melanin nanoparticles. (b) The DPPH scavenging rate of TP-Mg nanoparticles. (c) The PTIO scavenging rate of melanin nanoparticles in different pH conditions. (d) The PTIO scavenging rate of TP-Mg nanoparticles in different pH conditions. Sample size *n* = 3 for all experiments.

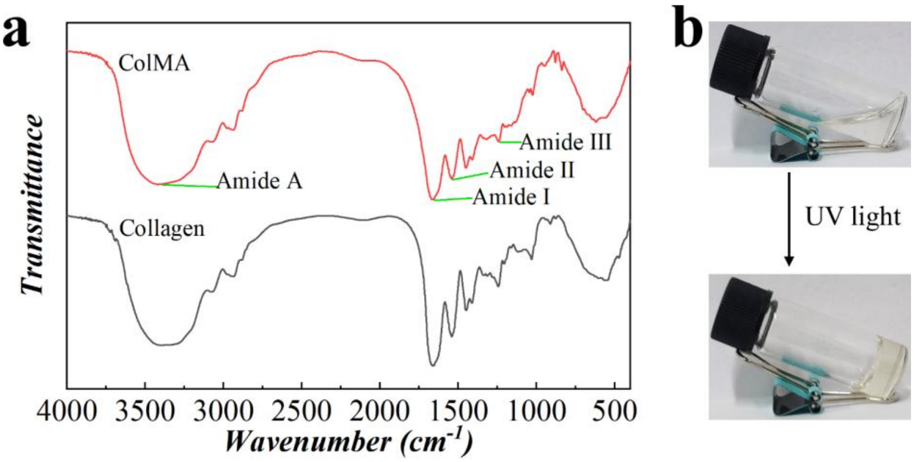

Figure S7. Fabrication of ColMA. (a) The FTIR spectrum of collagen and fabricated ColMA. (b) The optical images of ColMA sol and UV-polymerized gel.

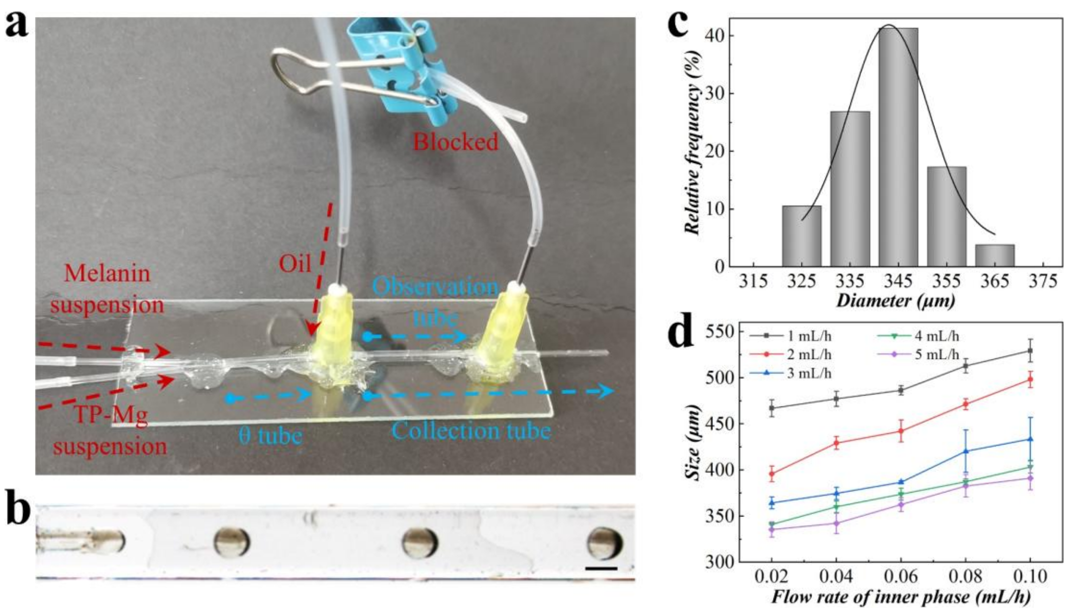

Figure S8. Microfluidics and characterization of Janus microparticles. (a) The image of

microfluidics device. (b) The real-time generation of Janus microdroplets in microfluidics device. (c) The size distribution of fabricated hydrogel microparticles in a typical experiment. (d) The relationship of droplets' size to flow rate of inner phase and outer phase. Scale bar is 400 μm in (b). Sample size $n \geq 50$.

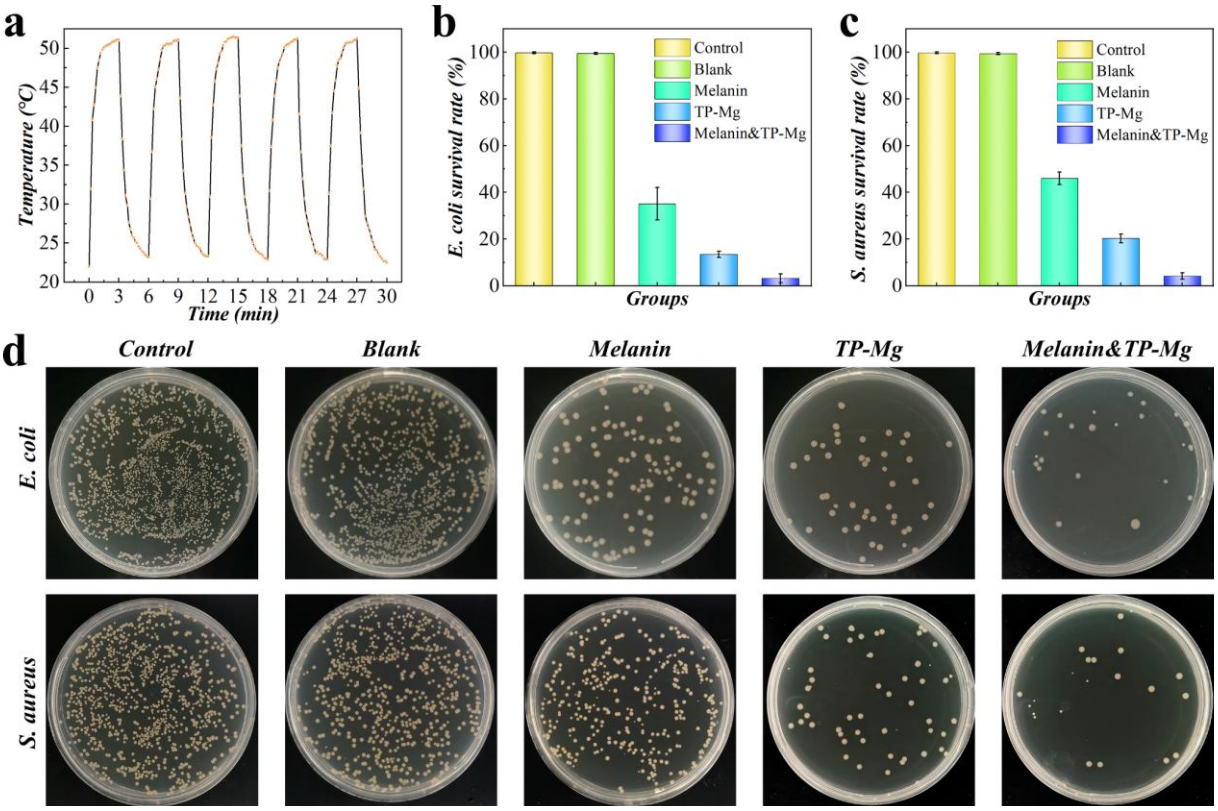

Figure S9. Heating stability and antibacterial test. (a) The cyclic test of NIR-responsive heating performance. (b) The survival rate of *E. coli* received different treatments. (c) The survival rate of *S. aureus* received different treatments. (d) The bacterial colony formed on LB agar plate in different groups. Sample size $n = 3$ for all experiment.

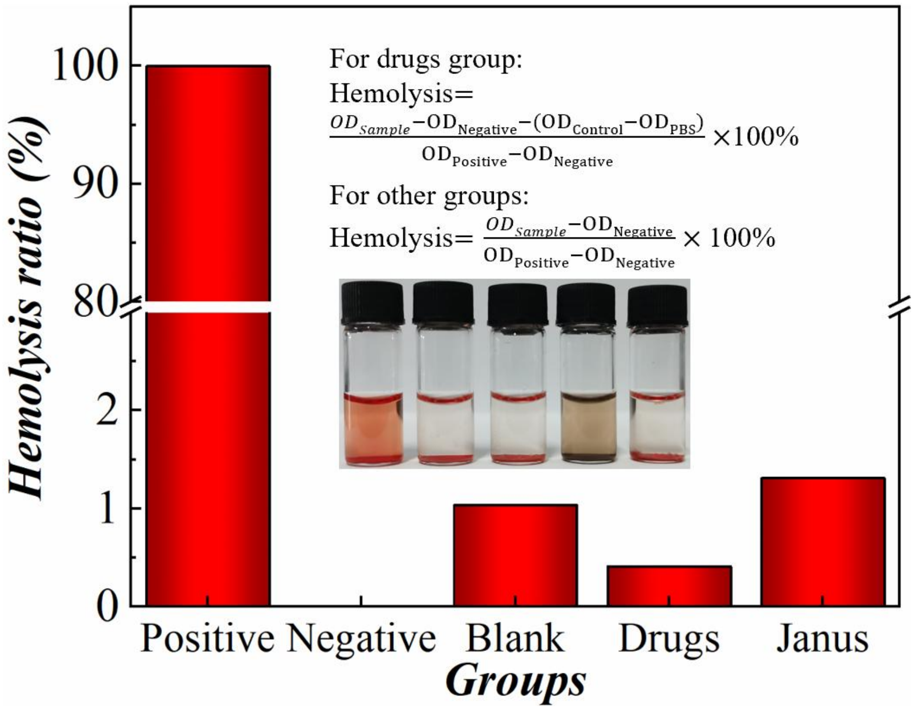

Figure S10. Blood compatibility result.

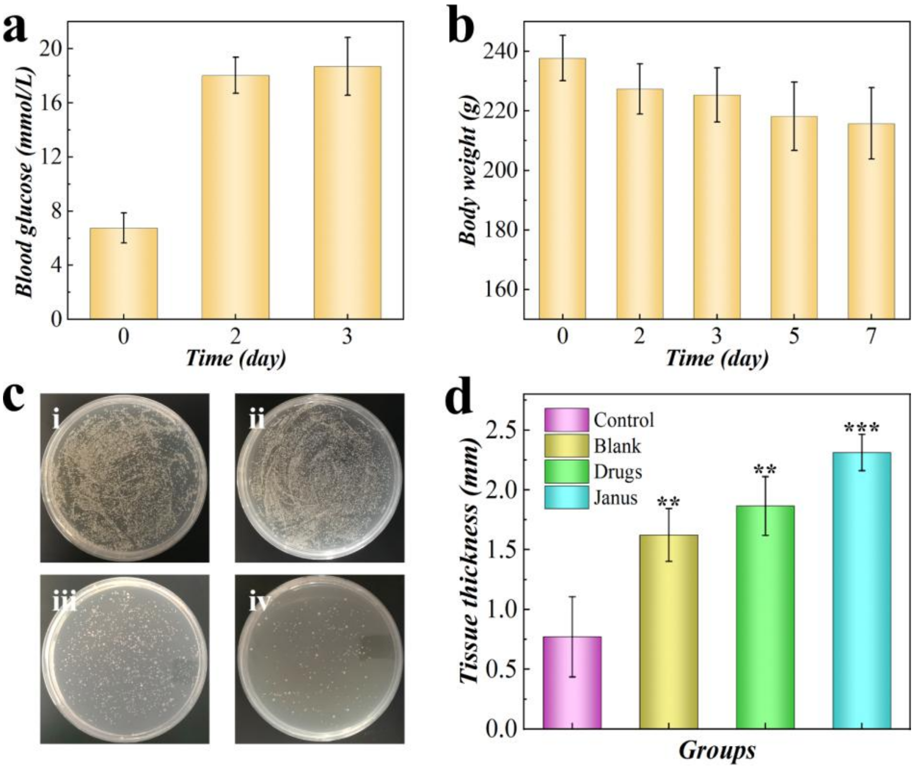

Figure S11. Diabetes induction and healing process evaluation. (a) The recorded blood glucose level. (b) The recorded change of body weight. (c) *Ex vivo* antibacterial test on agar plate coating of bacteria collected from wounds in different groups. (d) The thickness of

regenerated tissues in different wounds. Sample size $n = 12$ in (a-b) and $n = 8$ in (d). *$p < 0.05$, **$p < 0.01$, ***$p < 0.001$.

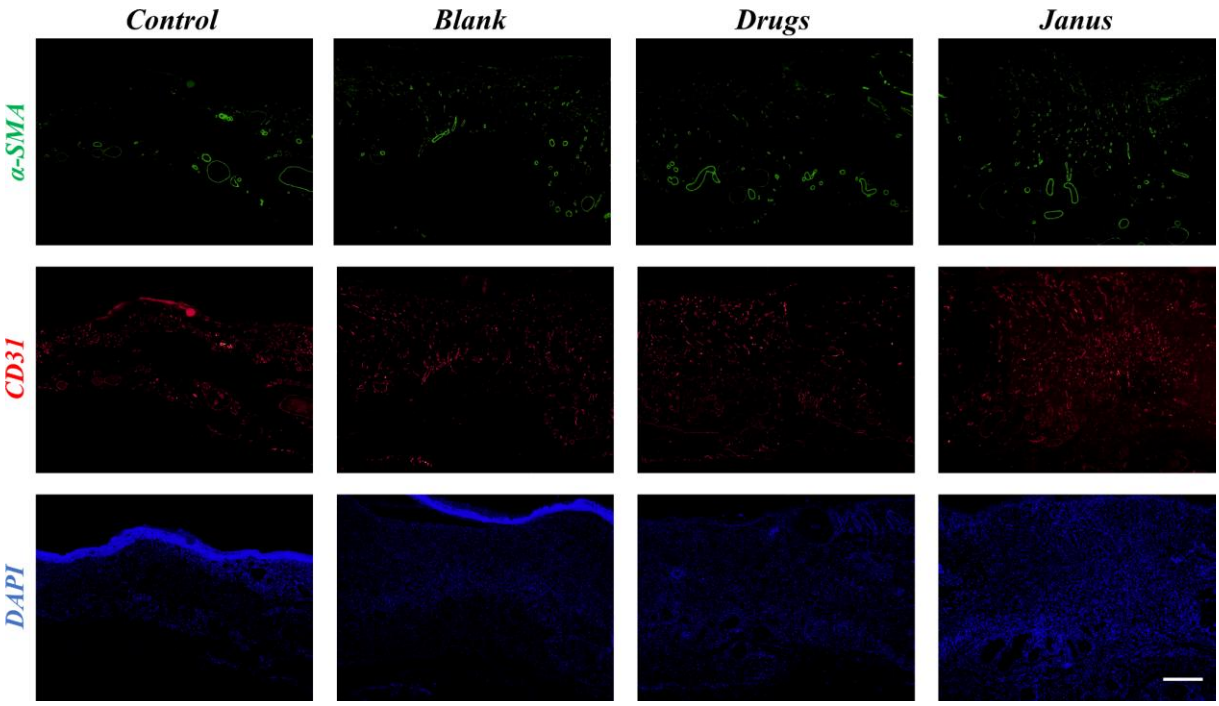

Figure S12. Fluorescent images of tissues stained by α-SMA, CD31 and DAPI in different groups. Scale bar is 400 μm.
